# Model-based optimization of controlled release formulation of levodopa for Parkinson’s disease

**DOI:** 10.1038/s41598-023-42878-5

**Published:** 2023-09-22

**Authors:** Yehuda Arav, Assaf Zohar

**Affiliations:** 1 Department of Applied Mathematics, Israeli Institute for Biological Research, PO Box 19, 7410001 Ness-Ziona, Israel; 2 Ness-Ziona, Israel

**Keywords:** Computational models, Drug delivery, Pharmaceutics, Parkinson's disease

## Abstract

Levodopa is currently the standard of care treatment for Parkinson’s disease, but chronic therapy has been linked to motor complications. Designing a controlled release formulation (CRF) that maintains sustained and constant blood concentrations may reduce these complications. Still, it is challenging due to levodopa’s pharmacokinetic properties and the notion that it is absorbed only in the upper small intestine (i.e., exhibits an “absorption window”). We created and validated a physiologically based mathematical model to aid the development of such a formulation. Analysis of experimental results using the model revealed that levodopa is well absorbed throughout the entire small intestine (i.e., no “absorption window”) and that levodopa in the stomach causes fluctuations during the first 3 h after administration. Based on these insights, we developed guidelines for an improved CRF for various stages of Parkinson’s disease. Such a formulation is expected to produce steady concentrations and prolong therapeutic duration compared to a common CRF with a smaller dose per day and a lower overall dose of levodopa, thereby improving patient compliance with the dosage regime.

## Introduction

Parkinson’s disease (PD) is a chronic neurodegenerative disorder caused by the progressive loss of dopamine-producing neurons in the brain^1^. The main symptoms of PD include resting tremor, rigidity, and slow movement (bradykinesia)^2^. While there is no known cure for PD, symptom management can help improve the patient’s quality of life^3^.

The most effective drug to control the symptoms of PD is levodopa, and oral delivery is the preferred route for its administration^2,4^. However, levodopa’s commercially available oral formulations produce pulsatile, non-physiological concentrations of levodopa in the blood^5^, and relief from PD symptoms for up to 4 h^6^. Over time, two significant problems can arise. First, the pulsatile concentrations of levodopa in the blood are considered to be one of the key factors that trigger the development of motor complications^2,7,8^ that emerge in about $50\%$ of the patients after 5 years of treatment^2,4,9,10^. Second, the clinical regime may require as much as 5-10 daily doses, given at specific timing^11,12^. Adhering to such a regime is difficult, and consequently, only $27-38\%$ of the doses are taken at the correct timing^13,14^.

Developing a new oral formulation of levodopa that produces sustained, smooth blood concentrations could address the limitations of current formulations^4^. A first step toward achieving this goal is to find, theoretically, the dose and release rate that would produce sustained therapeutic concentrations of levodopa in the blood. That is, identify the lowest possible dose that would maintain stable therapeutic concentrations of levodopa in the blood for the longest time.

Physiologically based pharmacokinetic modeling is a valuable approach to optimize drug absorption^15–21^. This approach involves modeling the physiological processes following oral administration using time-dependent differential equations, either partial or ordinary. Solving these equations makes it possible to quantify the contribution of different processes to the overall absorption process and consequently find optimal solutions. Guebila et al.^18^ used this approach to study the fluctuation of levodopa absorption under consideration of diet but did not consider sustained release formulations. Wollmer and Klein^20^ showed that using PD-specific in-vitro release models improves the accuracy of a whole-body pharmacokinetic model for levodopa. Cheung et al.^22^ and Zhang et al.^23^ used simplified kinetic models to provide general guidelines for designing controlled release formulation (CRF), i.e., a formulation that releases the drug over an extended period but does not address the specific traits of levodopa.

This work suggests guidelines for developing an improved CRF of levodopa. To accomplish this goal, we developed and validated a physiologically based mathematical model that focuses on the kinetics of levodopa absorption from its oral ingestion in CRF until it reaches the blood. Using the mathematical model, we were able to predict the mean concentration-time profile of levodopa in the blood based on the properties of the formulation (e.g., the dose and release rate) and to quantify the contribution of the physiological processes to the overall absorption process. The quantitative understanding of the absorption process allowed us to find the properties of a theoretical oral formulation expected to extend the time to deliver steady blood concentrations.

## Methods

In the following, we present a mathematical model for the absorption of levodopa following its administration in a CRF. We focus on CRF since such formulations are expected to prolong the absorption and produce smooth concentrations of levodopa in the blood^2,24^. However, clinical trials with commercially available CRFs, such as Sinemet CR, have not demonstrated a reduction in motor complications, which was attributed to erratic absorption that results in a pulsatile concentration of levodopa in the blood^4,5^. Hence, our first goal will be to use the model to understand the reason for their failure. With this understanding, we hope to suggest the properties of a putative improved CRF.

**Figure 1:**
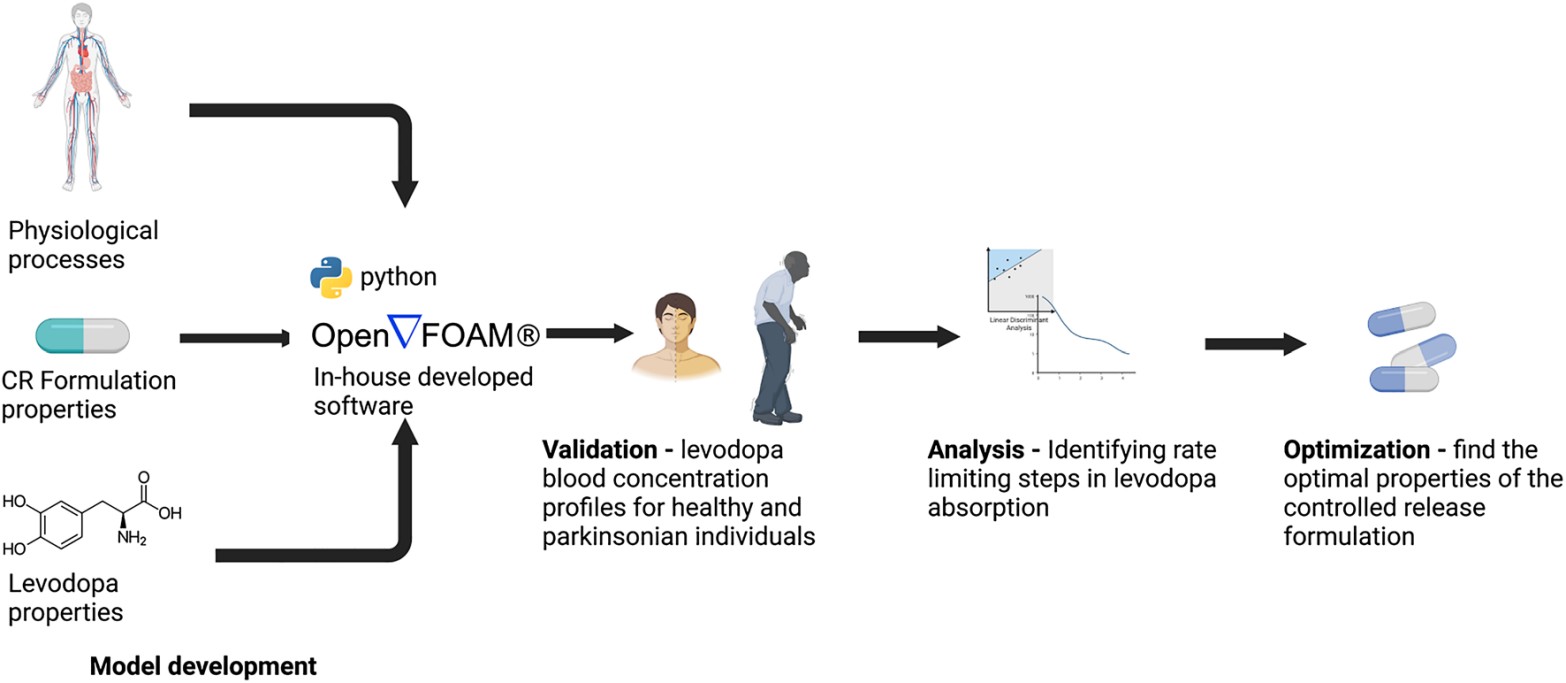
Schematic flow diagram of the model development and its usage to develop guidelines. Created with BioRender.com.

The model was developed based on the physiological processes, properties of the CRFs, and the properties of levodopa that were reported in the literature^15,16,25–35^. The model was validated by comparing its predictions to experimental results. In particular, we compare the model predictions for the blood concentrations of levodopa following administration of different CRFs to healthy and Parkinsonian individuals to the experimental blood concentrations taken from Arav^19^, Flashner et al.^32^, and Hammerstad^36^. Analyzing the model equations, we have identified the rate-limiting processes and used these insights to develop the guidelines for the optimal CRF. The workflow is illustrated in Fig. 1.

### Modeling the oral absorption of controlled release levodopa

**Figure 2:**
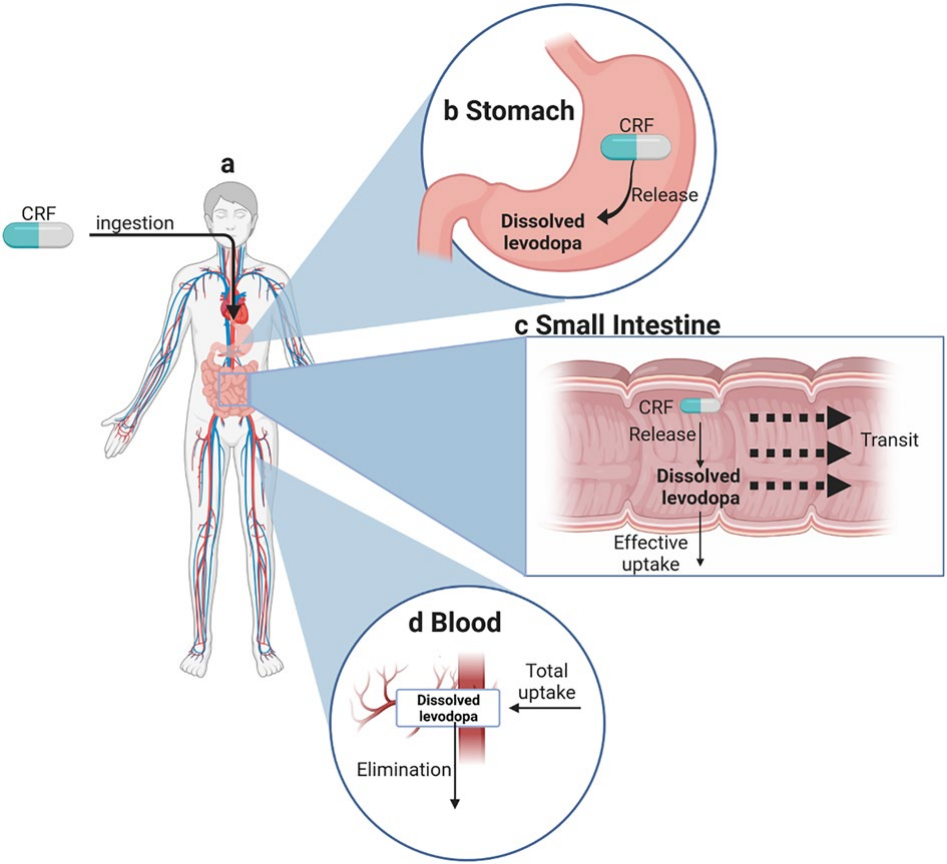
**(a)** The route of levodopa in the gastrointestinal tract following oral administration in CRF until it reaches the blood. **(b)** The processes that occur in the stomach. **(c)** The processes that occur in the small intestine. **(d)** The processes that occur in the blood. Created with BioRender.com.

Figure 2a depicts the route levodopa undergoes after oral administration in a CRF. Following ingestion, the CRF passes through the mouth cavity and the esophagus and reaches the stomach^37,38^.

#### The Stomach

A bean-shaped muscular bag that acts as a reservoir and regulates the transfer to the small intestine (SI) through the pylorus sphincter^16^. Figure 2b illustrates the processes that occur in the stomach. Upon reaching the stomach, the CRF begins to release levodopa. The CRF remains in the stomach for $\tau _{CRF-stomach}$ minutes before it passes into the SI.

The following ordinary differential equation describes the amount of levodopa in the CRF,

1 $$\begin{aligned} \frac{dA_{CRF}}{dt} = -\text {[Release](t)} \end{aligned}$$

where $A_{CRF}$ (mg) is the amount of levodopa in the CRF. The term [Release] ($\text{mg}\, \text{h}^{-1}$) represents the release rate of levodopa from the CRF. When the model describes the absorption process of levodopa following ingestion of an existing formulation, this term is determined from in-vitro experiments (see “Release from CRF” section below). In developing the guidelines, we will use the model to determine the desired release rate, as discussed in section ’Guidelines for designing levodopa CRF’ below.

The levodopa that was released is rapidly dissolved^26,39^, and emptied continuously to the SI. That is, the time scale of the dissolution process is much shorter than the time scale of the other processes in the stomach. Therefore, we consider the dissolution process as an instantaneous process. The concentration of dissolved levodopa in the stomach is determined by the release rate from the CRF (while it is in the stomach) and the transfer from the stomach to the SI (stomach emptying). After the CRF passes to the SI, the dissolved levodopa in the stomach ($C_{st}$) is determined only by the stomach emptying. Due to the stomach’s shape and size, we consider the stomach as a well-mixed compartment.

The ordinary differential equation gives the temporal change in the concentration of dissolved levodopa,

2 $$\begin{aligned} \frac{dC_{st}}{dt} = - \text {[Stomach emptying](t)} + {\left\{ \begin{array}{ll} \frac{\text {Release(t)}}{V_{st}} &{} t< \tau _{CRF-stomach} \\ 0 &{} t> \tau _{CRF-stomach} \end{array}\right. } \end{aligned}$$

where $V_{st}$ ($\text{cm}^3$) is the volume of the stomach, and [Stomach emptying] ($\text{mg}\, \text{h}^{-1}$) represents the transfer rate of the dissolved levodopa to the SI. Following Oberle et al.^28^, stomach emptying is modeled as a first-order process. However, dissolved levodopa can cause erratic gastric emptying^30,31,34,35,40,41^, that manifests by temporarily halting the emptying from the stomach (a ’lag’ in emptying). The erratic emptying exhibits high inter- and intra-subject variability and might not always exist. The mechanism that causes the lag in gastric emptying is not yet known^40^, and therefore we empirically incorporate the lag in the present model similarly to the empirical model of Ogungbenro et al.^41^. Specifically, the erratic emptying is described as a single lag that begins $\tau _{lag-start}$ minutes after ingestion, and lasts for $\tau _{lag-duration}$ minutes. Specifically,

3 $$\begin{aligned} \text {[Stomach emptying](t)} = {\left\{ \begin{array}{ll} \beta C_{st} &{} t \le \tau _{lag-start} \\ 0 &{} \tau _{lag-start}< t \le \tau _{lag-start} + \tau _{lag-duration} \\ \beta C_{st} &{} \tau _{lag-start} + \tau _{lag-duration}< t \\ \end{array}\right. } \end{aligned}$$

Where $\beta$ ($h^{-1}$) is the stomach emptying rate constant. The initial conditions of $A_{CRF}$ and $C_{st}$ depend on the properties of the CRF and are described in the section Release from CRF.

#### The Small Intestine (SI)

A long (280cm) and narrow tube where most drug and nutrient absorption occurs^16,38^. The SI is connected to the stomach by the pylorus sphincter on one end and to the large intestine through the ileocecal sphincter on the other. Anatomically, the SI is divided into three distinct regions: Duodenum (20 cm long), Jejunum (104 cm long), and the Ileum (156 cm long)^16^. Three structures amplify the surface area in contact with the intestinal fluid: large folds, finger-like projects (villi), and additional smaller protrusion (microvilli)^16,38^. The large folds are typically found between the mid-duodenum to mid-ileum and their size and density decrease along the SI. The amplification of the Villi, finger-like projections, and the microvilli to the surface area of the SI also decreases along its length (see supplementary information for further details).

The processes that take place in the SI are depicted in Fig. 2c. The dissolved levodopa and the CRF, which has been transferred from the stomach to the SI, are propelled toward the end of the SI through the contraction of its walls^25^. In the SI, the CRF continues to release levodopa at a similar rate to that in the stomach, which quickly dissolves^19,26^. Since the tablet’s dimensions are much smaller than the length of the SI, we describe the tablet as a point that releases levodopa in its location. The Large Neutral Amino Acid (LNAA) transporters actively transport the dissolved levodopa into the bloodstream^26,42,43^.

The SI is described as a continuous tube with spatially varying properties, such as the effective surface area, similar to several previous studies^15,16,19^. The concentration of the dissolved levodopa along the SI is determined by the stomach-emptying rate of dissolved levodopa, the release rate from the CRF, uptake to the blood by the LNAA transporters, and the intestinal transit. The partial differential equation determines the temporal and spatial distribution of the dissolved levodopa in the SI,

4 $$\begin{aligned} \frac{\partial C_{SI}(x,t)}{\partial t}&= \text {[Transit]}(x,t) - \text {[Effective uptake]}(x,t) \nonumber \\&\quad + {\left\{ \begin{array}{ll} 0 &{} t < \tau _{CRF-stomach} \\ \frac{\delta (x-CRF_l)}{\pi R_{SI}^2}\text {Release(t)} &{} t > \tau _{CRF-stomach} \end{array}\right. } \end{aligned}$$

Where *x* is the distance from the pylorus, $C_{SI}(x,t)$ (mg $\text{cm}^{-3})$ is the concentration of dissolved levodopa at point *x* in time *t*, $\delta$ ($\text{cm}^{-1}$) is Dirac’s delta function, $R_{SI}$ (*cm*) is the radius of the SI, and $CRF_l(t)$ is the location of the CRF along the SI,

5 $$\begin{aligned} CRF_l(t) = u\cdot(t-\tau _{CRF-stomach}) \end{aligned}$$

Where *u* (cm $\text{h}^{-1}$) is the mean convection velocity.

The [Transit] (mg $\text{cm}^{-3}$ $\text{h}^{-1}$) term describes the concomitant propulsion of dissolved levodopa by the SI walls toward its end and the mixing. Following Ho et al.^15^, the SI is described as a continuous tube. The propulsion of the dissolved levodopa toward the distal SI is described by a convection term of the mean velocity, while the mixing along the SI is described as a dispersion term (that is, an effective diffusion term). Specifically,

6 $$\begin{aligned} \text {Transit}(x,t) = \underbrace{\frac{\partial uC_{SI}}{\partial x}}_{Convection} \underbrace{-SP_{eff}\frac{\partial ^2 C_{SI}}{\partial x^2}}_{Mixing} \end{aligned}$$

where $SP_{eff}$ ($\text{cm}^{2}$ $\text{h}^{-1}$) is the effective longitudinal mixing rate of the SI. The negative sign of the mixing term denotes that the mixing flux is in the counter-gradient direction. Equation (4) is a partial differential equation and therefore require boundary conditions. These boundary conditions describe the flux of dissolved levodopa to the beginning of the SI from the stomach and the flux of dissolved levodopa from the SI to the large intestine at its distal end. Accordingly, the emptying from the stomach and the emptying to the large intestine is described as

7 $$\begin{aligned} \left. u\cdot C_{SI} - SP_{eff}\frac{\partial }{\partial x}C_{SI}\right| _{x=0}&= \frac{V_{st}\text {[Stomach emptying](t)}}{\pi R_{SI}^2} \end{aligned}$$

8 $$\begin{aligned} \left. u\cdot C_{SI} - SP_{eff}\frac{\partial }{\partial x}C_{SI}\right| _{x=L_{SI}}&= u^*\cdot C_{SI} \end{aligned}$$

where $L_{SI}$ (cm) is the length of the SI. The contents of the SI reside in the terminal SI for $\tau _{SI-Emptying}$ minutes before it is emptied to the colon^29^. Hence $u^*$ equals 0 before emptying commences and *u* when the SI is emptied (at $\tau _{SI-Cecum}$).

The [Effective uptake] (mg $\text{cm}^{-3}$ $\text{h}^{-1}$) describes the uptake of levodopa by the large amino acids transport system from the SI to the blood. It is generally thought that levodopa is absorbed only in the upper third of the SI (the Duodenum and upper Jejunum), known as the ’absorption window’^4,32,42,44–47^. However, Caramago et al.^42^ and Flashner et al.^47^ provided empirical evidence that levodopa absorption is comparable along the entire SI. The location of levodopa absorption within the SI is important because it affects the duration of absorption and therapeutic effects. We formulated two models to test the two hypotheses. Model A accounts for an ’absorption window’; Levodopa is effectively absorbed only from the upper third of the SI. In this model, the [effective uptake] term takes the form,

9 $$\begin{aligned} \text {[Effective uptake]}(x,t) = {\left\{ \begin{array}{ll} \text {[Uptake](x,t)} &{} x<\frac{L_{SI}}{3} \\ 0 &{} \text {Otherwise} \end{array}\right. } \end{aligned}$$

where the [Uptake] term describes the saturable uptake process of levodopa from the SI^26^,

10 $$\begin{aligned} \text {[Uptake]}(x,t) = \frac{2SA_{SI}}{R_{SI}}\frac{\phi _{max}C_{SI}}{K_m+C_{SI}} \end{aligned}$$

$SA_{SI}(x)$ is the amplification of the SI surface area due to the folds and the villi (see supplementary information for further details)^16^, $\phi _{max}$ (mg $\text{cm}^{-2}$ $\text{h}^{-1})$ and $K_m$ (mg $\text{cm}^{-3})$ are the maximal transport rate and the association constant of the LNAA.

Alternatively, model B accounts for absorption of levodopa along the entire SI. In this model, the [Effective uptake] term takes the form,

11 $$\begin{aligned} \text {[Effective uptake]}(x,t) = \text {[Uptake](x,t)} \end{aligned}$$

The unabsorbed levodopa and the CRF not degraded in the distal end of the SI, eventually pass into the colon, from which the absorption is negligible^18^.

#### Blood

The concentration of levodopa in the blood (Fig. 2d) is determined by the total uptake of dissolved levodopa along the SI, and the elimination and the distribution processes in the body^48,49^. Following Cedarbaum et al.^49^, we describe levodopa’s elimination and distribution processes in the body using a single-compartment model^50^. The ordinary differential equation describes the kinetics of the concentration of levodopa in the blood,

12 $$\begin{aligned} \frac{dC_{b}}{dt} = \frac{1}{V_d}\underbrace{\int _0^L\pi R_{SI}^2\text {[Effective uptake]}(x',t)dx'}_{\text {Total uptake}} - \underbrace{k\cdot C_b}_{\text {Elimination}} \end{aligned}$$

Where $V_d$ ($\text{cm}^3$) represents the volume of distribution of levodopa, *k* ($\text{h}^{-1}$) is the elimination rate from the body, and $C_b$ (mg $\text{cm}^{-3}$) is the blood concentration of levodopa.

### Release from CRF

This study aims to establish guidelines for the design of CRFs, specifically focusing on determining the [Release] term in Eq. (1). However, to validate the model and to determine whether or not levodopa has ’absorption window’ in the SI, we simulated two CRFs: Sinemet CR and the enteric-coated controlled release (ECCR) of Flashner et al.^32^. The modeling of these two CRFs consists of determining the [Release] rate and the initial conditions of $A_{CRF}$ and $C_{st}$.

Sinemet CR releases levodopa in first-order kinetics^33^,

13 $$\begin{aligned} \text {Release(t)} = \alpha _{Sinemet}\cdot A_{CRF} \end{aligned}$$

Where $\alpha _{Sinemet}$ ($\text{h}^{-1}$) is the erosion rate coefficient of Sinemet CR. It has been reported that in many monolithic matrix controlled release tablets, such as Sinemet CR, there is an initial large release of the drug (’burst release’) before the release rate reaches a stable profile. This phenomenon is often observed in the literature^33,51^; hence, we have incorporated such a process into the model. The initial conditions of $A_{CRF}$ and $C_{st}$ incorporate the ’burst’ phase into the model. Specifically,

14 $$\begin{aligned} A_{CRF}(0)&= D - B \end{aligned}$$

15 $$\begin{aligned} C_{st}(0)&= \frac{B}{V_{st}} \end{aligned}$$

Where *D* (mg) is the administrated dose, and *B* (mg) is the burst size.

The ECCR is designed to delay the release of levodopa to approximately an hour after the tablet leaves the stomach^32^. Once the release began, the release rate of ECCR followed a first-order kinetics^32^. Hence the [Release] term is,

16 $$\begin{aligned} \text {Release(t)} = {\left\{ \begin{array}{ll} 0 &{} t < \tau _{ECCR-delay} \\ \alpha _{ECCR}\cdot A_{CRF} &{} t > \tau _{ECCR-delay} \end{array}\right. } \end{aligned}$$

Where $\alpha _{ECCR}$ ($\text{h}^{-1}$) is the erosion rate coefficient of ECCR, and $\tau _{ECCR-delay}$ is the time the ECCR commences the release.

### Model parameters

The model was employed to predict levodopa kinetics in the blood of both healthy individuals and Parkinsonian patients. All the model parameters used to describe healthy patients were compiled from various sources in the literature^15,16,25–33^ (Table 1), except for *B* of Sinemet CR. This parameter was obtained by fitting to the observed blood concentrations of healthy subjects at 30 min following administration of Sinemet CR 100/25 mg.

**Table 1:** The parameters used in the levodopa simulations.

Parameter name	Value	Reference	Comment
Stomach			
Volume ($V_{st}$)	200$\,\text{cm}^3$	^28^	
Lag duration ($\tau _{lag-duration}$)	0.5 h	^31^	
Healthy subjects			
Stomach emptying ($\beta$)	$8.31\,\text{h}^{-1}$	^28^	$t_{1/2}$ of 5min
Lag start ($\tau _{lag-start}$)	$0.5\,\text{h}$	^31^	
Stomach residence time ($\tau _{CRF-stomach}$)	$1\,\text{h}$	^25,52^	
Parkinsonian subjects			
Stomach emptying ($\beta$)	$1.38\,\text{h}^{-1}$		See text
Lag start ($\tau _{lag-start}$)	$1\,\text{h}$		See text
Stomach residence time ($\tau _{CRF-stomach}$)	$1.5\,\text{h}$		See text
Small intestine (SI)			
Length ($L_{SI}$)	282 cm	^38^	
Radius ($R_{SI}$)	2.5 cm	^16,38^	
Transit velocity (*u*)	94 $\text{cm}\, \text{h}^{-1}$	^53^	Transit time 3 h
Spread coefficient ($SP_{si}$)	1800 $\text{cm}^2\, \text{h}^{-1}$	^53^	
Surface Area amplification ($SA_{SI}$)		^16^	
LNAA transport rate ($\phi _{max}$)	5.868 $\text{mg}\, \text{h}^{-1} \text{cm}^{-2}$	^54^	Fitted from d
LNAA $K_m$ ($K_{m}$)	$1.47\,\text{mg}\, \text{cm}^{-3}$	^26^	7.5 $\text{mM}$
Healthy subjects			
SI emptying time ($\tau _{SI-Cecum}$)	5.5 h	^29^	Terminal SI residence of 1.5 h
Parkinsonian subjects			
SI emptying time ($\tau _{SI-Cecum}$)	6 h	^29^	Terminal SI residence of 1.5 h
Body			
Volume of distribution ($V_d$)	$63,000\,\text{cm}^3$	^49^	0.9 $\text{L}\, \text{kg}^{-1}$
Elimination rate constant (*k*)	$0.4608\, \text{h}^{-1}$	^27^	$t_{1/2}$ of 1.5 h
Formulation			
Sinemet CR			
Dosage (*D*)	$100\,\text{mg}/200\,mg$	^33^	
Commence of release	$0\,\text{h}$	^33^	
First order release ($\alpha _{Sinemet}$)	$0.23\,\text{h}^{-1}$	^33^	$t_{1/2}$ of 3 h
Dose dump (’burst’, *B*)	$60\,\text{mg}$		See text
Enteric coated CR			
Dosage (*D*)	$200\,\text{mg}$		
Release commence $\tau _{ECCR-delay}$	$2\,\text{h}$	^32^	1 h after stomach emptying
First order release ($\alpha _{ECCR}$)	$0.23\,\text{h}^{-1}$	^32^	$t_{1/2}$ of 3 h
Dose dump (’burst’,*B*)	0*mg*	^32^	

Previous studies^48,55–57^ have shown that rate constants for these processes are similar for healthy and parkinsonian patients with the exception that stomach emptying and intestinal motility are retarded by PD. We have adjusted the model parameters determining gastric emptying and gastrointestinal motility: the stomach emptying $t_{1/2}$ and the residence time of Sinemet CR in the stomach was fitted to the experimental results of Sinemet CR 100/25mg (see supplementary results).

### Numerical methods

The system of ordinary and partial differential Eqs. (1–16) were solved numerically using in-house software (available at https://github.com/yehudarav/levodopaAbsorption). The software was developed using the open Field Operation and Manipulation (OpenFOAM) framework (version extend-5)^58^, which extends the C++ programming language to include tensor calculus. The temporal derivations were solved using a first-order implicit scheme. The partial differential equation (Eq. 4) was solved using the finite volume method.

Analysis of the results, Eqs. (17–22), and the optimization methods were implemented in Python 3.9.

ChatGPT, a language model developed by OpenAI^59^, was used to identify and rectify grammatical errors, improve sentence construction, and enhance overall readability.

## Results

### Testing model A and model B

Before we use the model, it is necessary to ascertain its ability to predict the overall absorption process of levodopa in healthy and Parkinsonian subjects following administration in a commercially available CRF. Due to the conflicting data in the literature on the absorption sites of levodopa along the SI, we developed two models: one that considers an ’absorption window’ (model A) and one that considers absorption along the entire SI (model B). We now test the two models by comparing their predictions to the blood concentrations of levodopa obtained following oral administration of levodopa in a CRF.

**Figure 3:**
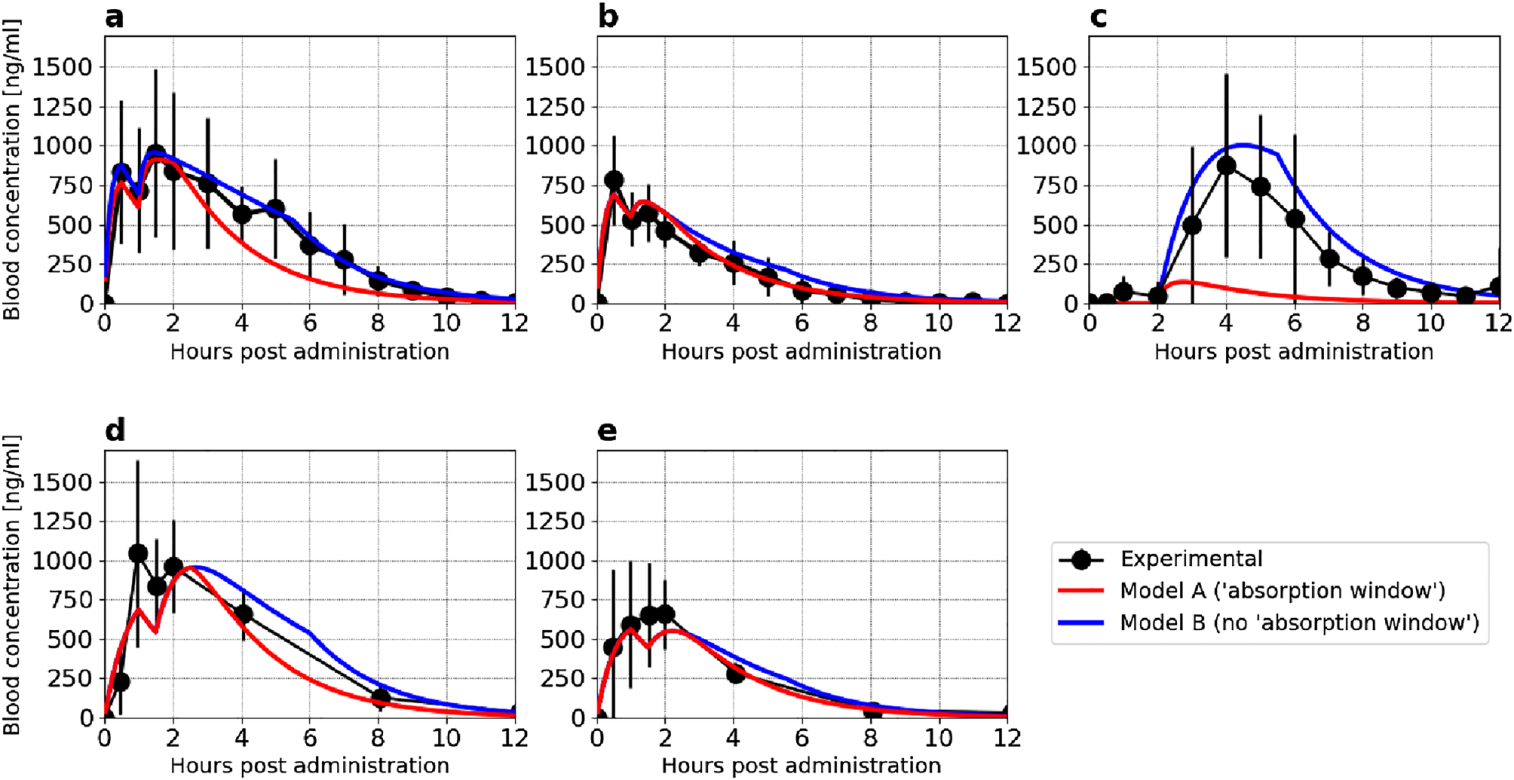
Comparing experimental and model prediction of blood concentration of levodopa. (**a**) Sinemet CR 200/50 mg in 7 healthy fasting subjects, (**b**) Sinemet CR 100/25 mg in 8 healthy fasting subjects, (**c**) Enteric-coated controlled release 200 mg in 12 healthy fasting subjects, (**d**) Sinemet CR 200/50 mg in 9 Parkinsonian fasting patients, and (**e**) Sinemet CR 100/25 mg in 9 Parkinsonian fasting patients. Data for Sinemet CR in healthy and Parkinsonian patients was taken from Arav et al.^19^ and Hammerstad1 et al.^36^, respectively. Data for enteric-coated controlled release was taken from Flashner et al.^47^.

We begin by comparing the model predictions to the experimental blood concentrations of levodopa following administration of Sinemet CR 200 mg to healthy fasting subjects (Fig. 3a black circle line, taken from Arav^19^). The mean experimental concentrations reach a maximum within 3 h and exhibit a double peak. The characteristics of the mean concentrations reflect the kinetics of the individuals as the rise, the location, and the amplitude of the double peaks and the decay are similar to those observed in the individuals (Fig. S1, supplementary information). The occurrence of a double peak in levodopa plasma concentrations has been previously reported^30,31,34,40,41^ and was attributed to the presence of dissolved levodopa in the stomach, which interferes with gastric emptying^30,31^. The mechanism of interference is not entirely understood^40^, and exhibits large variability^30,31^; in our dataset 5 individuals out of 7 exhibits a double peak (Fig. S1, supplementary information).

To test the model prediction for Sinemet CR 200 mg, we solve model A and model B equations using parameters reported in the Method section (Table 1). Figure 3a compares the predictions of model A (red) and model B (blue) to experimental data. As shown, the predictions of both models align well with the experimental mean blood concentrations during the first 2 h after administration, capturing the rise, location, and magnitude of the two peaks. Consistently with Robertson et al.^30,31^, the model predicts that the stomach emptying process contributes significantly to the high variability seen during the first 2 h (Fig. S4, supplementary information). At later times (3 h after administrations), model A underestimates the levodopa concentration in the blood starting 2 h after taking Sinemet CR 200 mg, while model B captures the kinetic in the blood well. The overall absorption (bioavailability) of Sinemet CR 200 mg is under-predicted by model A, while the prediction of model B is within the range reported in the literature. Specifically, the predicted bioavailability of model A and model B is $51\%$ and $77\%$, respectively, and the reported range is 71–78%^24,60^. These results suggest that a substantial portion of Sinemet CR 200 mg, approximately 20–25%, is absorbed in the lower two-thirds of the SI.

Figure 3b depicts the experimental (black circle line, taken from Arav et al.^19^) and the predictions of model A (red solid line) and B (blue solid line) following the administration of Sinemet CR 100mg to healthy fasting subjects. The mean concentration profile exhibits a double peak and obtains the maximal concentrations after 30min. As mentioned above, a double peak is attributed to dissolved levodopa in the stomach and is a highly variable process. Indeed, only half of the individuals exhibit a double peak (Fig. S2, supplementary information). However, since the double peak is seen in the mean concentrations, we present the simulation results with the lag in stomach emptying. As illustrated, both models align well with the experimental data, capturing the temporal patterns, peak locations, and decay of levodopa in the bloodstream. As seen, both models capture well the kinetics of the levodopa in the blood. Specifically, the rise, the location of the peaks, and the decay. This finding suggests that a negligible portion of Sinemet CR 100mg is absorbed in the lower two-thirds of the SI.

Figure 3c depicts the experimental (black circle line, taken from Flashner et al.^32^) and the predictions of model A (red solid line) and B (solid blue line) following administration of enteric-coated controlled release (ECCR) reported in Flashner et al.^47^. The ECCR was designed to release levodopa only in the lower Jejunum and the Ileum. As seen, the measured mean blood concentration profile of levodopa begins its rise 2 h after administration and exhibits a single peak after 4 h. The mean concentration profile reflects qualitatively the kinetic patterns observed in the individual subjects (see supplementary information for further details). Model A and model B predictions were obtained using the ECCR release term in Eq. (1) corresponding to the ECCR tablet (see “Methods” Section for further details). Model A underestimates levodopa’s experimental blood concentrations, while model B aligns well with the experimental data. These results prove that levodopa is absorbed well along the entire SI.

The results above demonstrate that the proposed model accurately predicts the concentration-time profile of levodopa in the blood following the administration of Sinemet CR to healthy subjects. However, previous studies have reported that patients with PD experience stomach emptying and intestinal motility delays, which may affect levodopa absorption’s kinetics^48,55–57^. Other pharmacokinetic aspects of levodopa are not impacted by PD^48,55–57,61^. To investigate this, we compared the experimental results to the model predictions of the concentration-time profile of levodopa following the administration of Sinemet CR to Parkinsonian patients. The mean blood concentrations of levodopa following administration of Sinemet CR 200 mg to Parkinsonian patients, as reported in Hammerstad et al.^36^, are depicted in Fig. 3d (black circle line). The mean blood concentration of levodopa in Parkinsonian subjects also exhibits a double peak with a similar amplitude to the peak obtained by healthy subjects, but it is delayed by approximately 30min (compare to Fig. 3a). In addition, the first peak is slightly higher than the second, albeit a large variation is reported. To predict the mean blood concentration of levodopa in Parkinsonian patients, we modified the model by lengthening the residence time of the CRF in the stomach and slowing its emptying rate (see Table 1 for details). Model A and model B predictions are depicted in Fig. 3d (red and blue solid lines, respectively). Both models capture the main features of levodopa’s kinetics in the blood of Parkinsonian patients, including the rise and decay kinetics and the location of the double peak. Both models slightly underestimate the mean levodopa concentration in the blood 1 hour after taking Sinemet CR 200 mg. However, the measured blood concentrations exhibited a large standard deviation, indicating that a highly variable process determined the blood concentrations. Model A predicts a slightly faster decline in the blood concentration of levodopa than model B, 2 h after administration. Nevertheless, both models provide a good description of the data. These results also suggest that a smaller fraction of the dose is absorbed in the lower SI of Parkinsonian patients compared to healthy subjects, due to the slower motility in the gastro-intestinal tract.

Figure 3e depicts the experimental results (black circle line, taken from Hammerstad et al.^36^) following the administration of Sinemet CR 100mg to Parkinsonian patients. The maximal blood concentrations are obtained within 2 h after administration. The concentrations are comparable to the concentrations obtained following administration to healthy subjects (Fig. 3b, black circle line), albeit the results does not exhibit a double peak, but have a large inter-individual variation. The absence of a lag could result from a small sample of individuals, or an artifact of the averaging. We have included a lag in these simulations, but we note that removing the lag produces a single peak (see supplementary information or details). Nevertheless, the predictions of Model A (Fig. 3e, red solid line) and model B (Fig. 3e, blue solid line) capture well the kinetics of the measured levodopa blood concentrations.

We conclude that levodopa is absorbed well along the entire SI and does not exhibit an ’absorption window’ and that model B predicts the kinetics of levodopa in the blood following a CRF for healthy and Parkinsonian patients. Hence, we will refer only to model B when developing the guidelines in the following.

### Rate-limiting processes in absorption following administration of Sinemet CR

As a preliminary step in developing the guidelines, we employ the model to gain insight to the rate-limiting processes in levodopa absorption and how the properties of Sinemet CR 200 mg influence levodopa blood concentrations in Parkinsonian patients (Simulation 3d). This analysis will allow us to identify the necessary improvements to the CRF.

**Figure 4:**
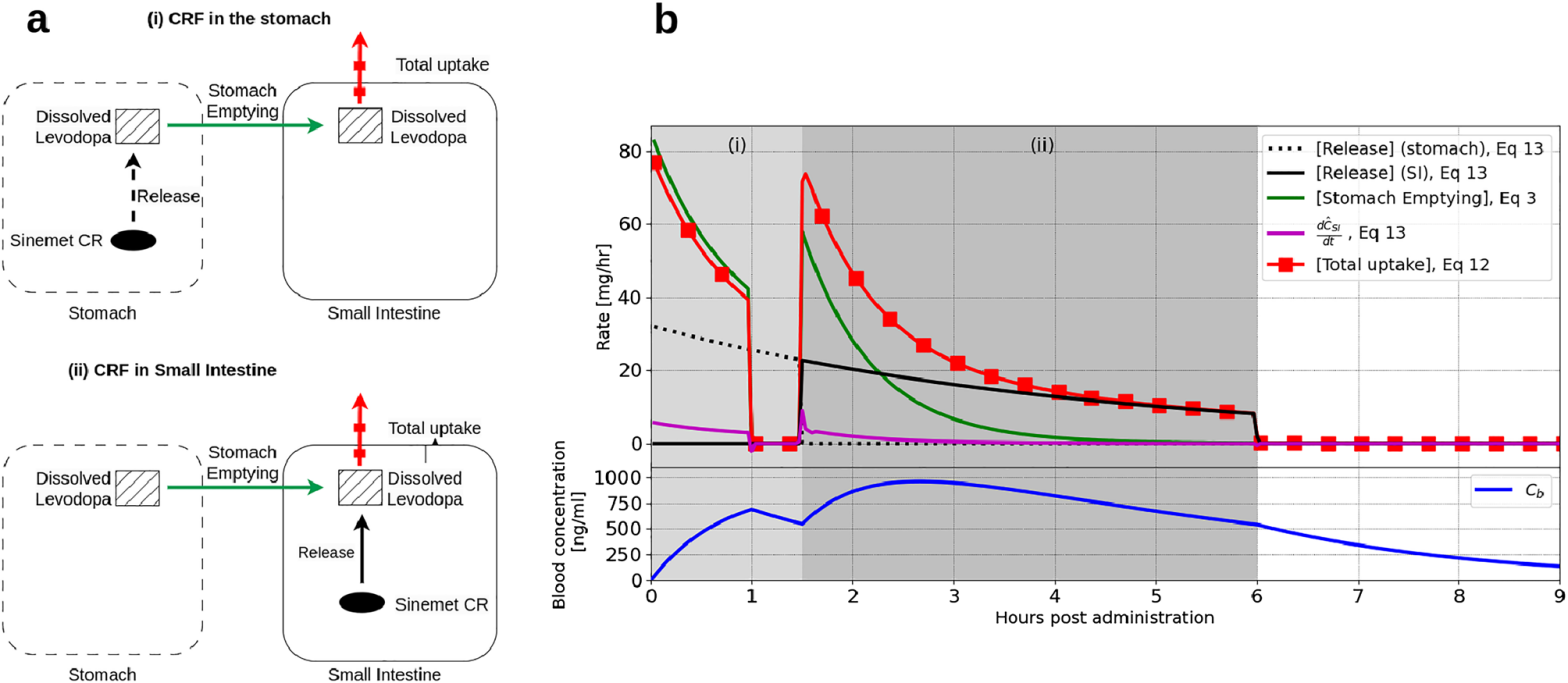
(**a**) Schematic representation of the absorption processes when Sinemet CR is in the stomach (i) and in the SI (ii). (**b**) Simulation results depicting the rates of the absorption processes and the corresponding levodopa concentration in the blood following administration of Sinemet CR 200 mg to Parkinsonian patient (Simulation 3d). The shaded areas (i) and (ii) refer to the time that Sinemet CR is in the stomach and the SI, respectively.

We first recall that the concentration of levodopa in the blood is determined by the balance between the uptake from the entire SI ([Total uptake] term, Eq. 12) and the elimination ([Elimination] term, Eq. 12). To determine how release from Sinemet CR determines the [Total uptake] term, we spatially integrate the equation for $C_{SI}$ (Eq. 4). While Sinemet CR is in the stomach, the schematics of the absorption process are illustrated in Fig. 4a-(i), and the corresponding equations are:

17 $$\begin{aligned} \frac{dC_{st}}{dt}&= - \text {[Stomach emptying]} + \frac{\text {[Release]}}{V_{st}} \end{aligned}$$

18 $$\begin{aligned} \frac{d\hat{C}_{SI}}{dt}&= \frac{1}{\pi R_{SI}^2}\left( V_{st}\text {[Stomach emptying]} - \text {[Total uptake]}\right) \end{aligned}$$

19 $$\begin{aligned} \frac{dC_{b}}{dt}&= \frac{\text {[Total uptake]}}{V_d} -\underbrace{k\cdot C_b}_{\text {Elimination}} \end{aligned}$$

where $\hat{C}_{SI}=\int _0^L C_{SI} dx$, the [Total uptake] taken from Eq. 12, and $u^*$ in Eq. 8 is zero before the emptying of the SI ($t<\tau _{SI-Cecum}$). The equation for $C_b$ (Eq. 12) is repeated here as Eq. 19 for clarity.

The schematics of the absorption process after Sinemet CR leaves the stomach and before the emptying of the SI, are illustrated in Fig. 4a-(ii). The equations obtained after integration along the SI in this case are:

20 $$\begin{aligned} \frac{dC_{st}}{dt}&= - \text {[Stomach emptying]} \end{aligned}$$

21 $$\begin{aligned} \frac{d\hat{C}_{SI}}{dt}&= \frac{1}{\pi R_{SI}^2}\left( V_{st}\text {[Stomach emptying]} + \text {[Release]} - \text {[Total uptake]}\right) \end{aligned}$$

22 $$\begin{aligned} \frac{dC_{b}}{dt}&= \frac{\text {[Total uptake]}}{V_d} -\underbrace{k\cdot C_b}_{\text {Elimination}} \end{aligned}$$

Using the model, we calculated the [Stomach emptying], [Release], and [Total uptake] terms following the administration of Sinemet CR 200 mg to Parkinsonian patients (Fig. 4b). The results of the model represent the mean case, and therefore, we anticipate that the general conclusions will be similar for individuals, although at different time points.

While Sinemet CR resides in the stomach (the first 1.5 h after administration in the simulation), the absorption follows the scheme illustrated in Fig. 4a-(i). During that time, the [Total uptake] almost coincides with the [Stomach emptying] term (compare the green and red square lines, Fig. 4b). Hence, stomach emptying is the rate-limiting step of levodopa absorption, and lags in stomach emptying result in lags in absorption that translate to troughs in the levodopa concentration in the blood. This is seen, for example, between 1 and 1.5 h after administration (blue line, Fig. 4b). The trending effect of stomach emptying on the blood concentrations of levodopa is further demonstrated in Fig. S4 in the supplementary.

After Sinemet CR passes to the SI, the absorption scheme changes to Fig. 4a-(ii). Initially (between 1.5 and 3 h after administration in the simulation), the [Stomach emptying] has the same order of magnitude as the [Release] term, and therefore, the absorption is determined by the two processes concomitantly. Hence, during that time, lags or delayed emptying from the stomach is expected to affect levodopa plasma concentrations, but to a lesser degree than phase (i).

Approximately 3–4 h after administration, the [Total uptake] coincides with the [Release] term (compare the black and red square lines, Fig. 4b) and $\frac{d\hat{C}_{SI}}{dt}\approx 0$ (magenta line, Fig. 4b). This can be attributed to two reasons. Firstly, all the dissolved levodopa was emptied from the stomach, and the CRF in the SI became the sole source of levodopa. Secondly, the maximal uptake rate exceeded the release rate, resulting in the absorption rate being equal to the release rate from Sinemet CR. After Sinemet CR is emptied into the large intestine, the [Total uptake] term decreases to zero.

Two intermediate conclusions can be drawn from the results of Fig. 4:

1. Rapid release of levodopa in the stomach produces a fast significant increase in levodopa blood concentrations. Nonetheless, lags in stomach emptying can arise as the dissolved levodopa is being emptied, leading to pulsatile blood concentrations. These are considered to be one of the key factors that trigger the development of motor complications^2,7,8^.
2. After levodopa was emptied from the stomach, the release rate of levodopa from the CRF primarily controls the absorption rate of levodopa released in the SI. Hence, maintaining a constant release rate throughout the entire SI would lead to stable blood concentrations of levodopa.

### Guidelines for designing levodopa CRF

The preliminary conclusions of the previous section indicate that it would be preferable to avoid releasing levodopa in the stomach. Instead, the emphasis should be on developing a formulation that rapidly transitions to the small intestine, releasing levodopa consistently upon entering the Duodenum and throughout its passage. The absorption from such a formulation is illustrated in Fig. 4a-(ii) and described with Eqs. (20–22).

One possible implementation of the aforementioned properties is utilizing a multi-particulate extended-release formulation (MPERF). In such formulations, each particle can be coated with an enteric coating to prevent the premature release of levodopa in the stomach. Furthermore, due to their size, these particles are not retained in the stomach^62^. An example of a product based on this technology is Rytary, an extended-release formulation for levodopa^63^. The guidelines presented in this study will outline the overall characteristics and requirements of such a formulation. However, the specific implementation details of this formulation are beyond the scope of the present research.

The characteristics of the putative CRF depend on the initial levodopa blood concentrations of the patient before administration. When levodopa blood concentrations are low, typically with a formulation taken in the morning, it is necessary to increase the concentration rapidly (the ‘loading dose’) and then maintain the concentrations at the therapeutic levels (‘maintenance dose’). Alternatively, when the initial levodopa blood concentrations are moderate, as would be expected for doses taken throughout the day, the formulation should contain a smaller fraction of the ’loading dose’.

We propose two variations of the MPERF formulation: the morning and late-day formulations. Each of these formulations consists of particles coated with two distinct types of coatings. The first type of particles releases levodopa upon reaching the Duodenum, serving as the loading dose. The second type, designed with an extended-release coating, constitutes the maintenance dose. Both the morning and late-day formulations include these two types of particles, with their proportions varying between the two formulations.

The following describes how to estimate the loading and maintenance doses. In the next section, we provide these values for the morning and late-day formulations for different stages of PD and demonstrate their effectiveness.

The maintenance dose can be implemented as particles that release levodopa at a constant rate in the SI. We make the assumption that the rate of levodopa release is directly proportional to the fraction of the dose that has been emptied from the stomach into the SI. The MPERF particles are emptied with the stomach content due to their size. Consequently, the fraction of the particles released from the stomach is obtained by integrating Eq. (20), and replacing $C_{st}$ with the desired release rate,

23 $$\begin{aligned} \text {[Release]} = \text {[Release}_{M}]\left( 1-e^{-\beta t}\right) \end{aligned}$$

Where [Release$_{M}$] is the desired release rate in the SI. The maintenance dose, $D_M$ is obtained by integrating the release term over time.

To determine [Release$_{M}$], we consider later time points after all the stomach content was released to the SI (e.g., $t > \frac{4}{\beta }$). At this time, [Stomach emptying]$\approx 0$ and the [Total uptake] term in Eq. (21) equals [Release]$_{M}$ since $\frac{d\hat{C}_{SI}}{dt}=0$ (Fig. 4b). Using Eq. (22), we find the desired release rate to be,

24 $$\begin{aligned} \text {[Release}_{M}] = k\cdot C_{target}\cdot V_d \end{aligned}$$

where $C_{target}$ are the desired blood concentrations.

The loading dose can be implemented as particles that release their content upon reaching the Duodenum. Hence, the [Stomach emptying] term describes their arrival rate into the SI. Solving Eq. (20), the [Stomach emptying] rate is,

25 $$\begin{aligned} \text {[Stomach emptying]} = D_L\beta e^{-\beta t} \end{aligned}$$

where $D_L$ is the amount of levodopa in the loading dose.

Given that the objective of the loading dose is to rapidly increase levodopa blood concentrations following administration, our next step is to determine the appropriate value of $D_L$ that would result in the desired $C_{target-loading}$ after 30 minutes. To accomplish this, we minimize the following equation:

26 $$\begin{aligned} \min _{D_{L}}&\left\{ (C_b(30min,D_{L})-C_{target-loading})^2\right\} \\&\text {with} \nonumber \\&C_b(0min)=C_{initial} \nonumber \end{aligned}$$

$C_{target-loading}$ is the desired loading concentration after 30 minutes, and $C_{initial}$ is the levodopa concentration in the blood when the dose is administrated. The $C_b$ is calculated from Eq. (22), using Eqs.(23 and 25), and recalling that [Total uptake]=[Stomach emptying]+[Release] (Fig. 4b).

### The suggested MPERF for different stages of PD

**Figure 5:**
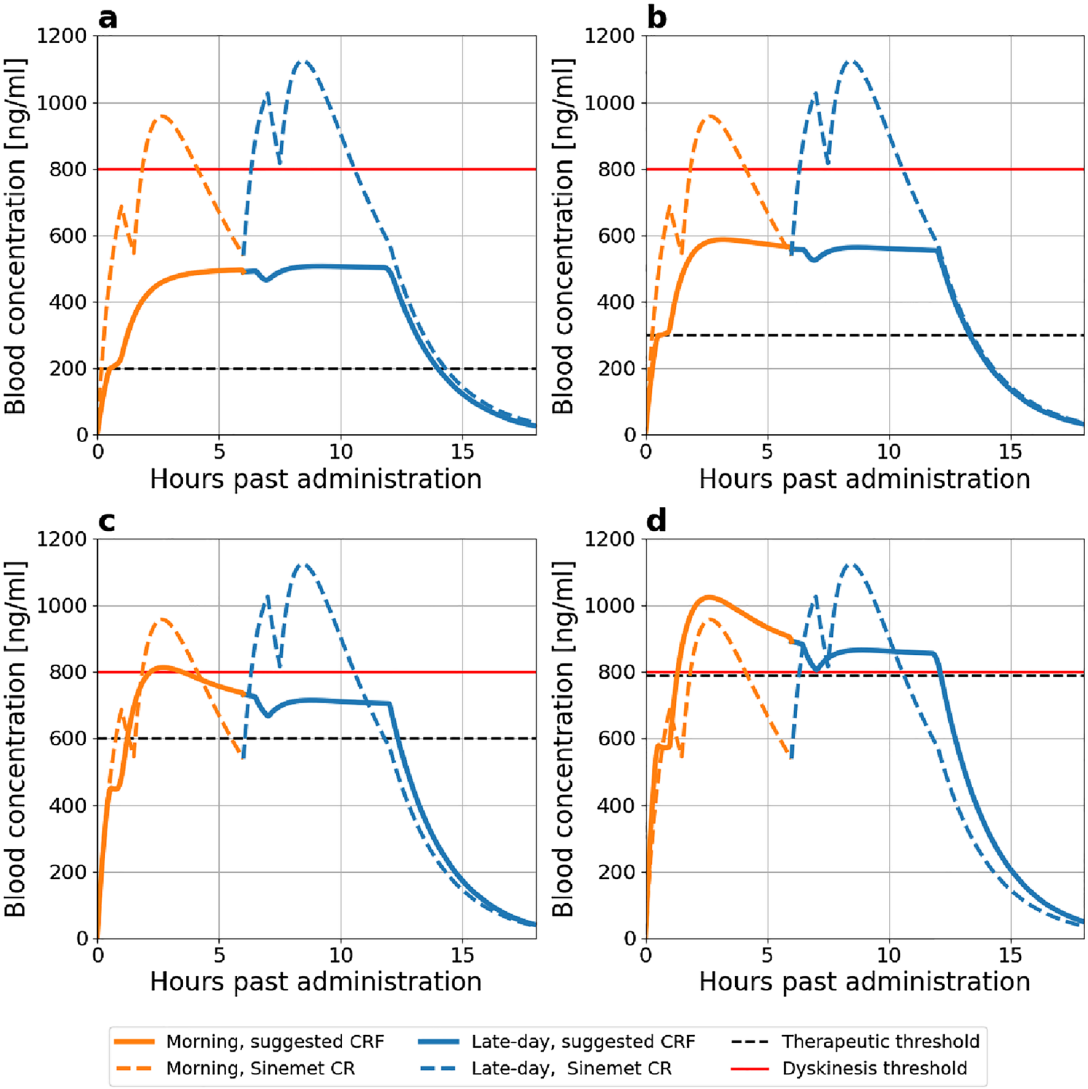
The predicted blood concentration of levodopa from the suggested morning and later-day MPERF detailed in Table 2 contrasted with 2 Sinemet CR 200/50 mg doses. (**a**) H &Y stage I, (**b**) H &Y stage II, (**c**) H &Y stage III, and (**d**) H &Y stage IV. Therapeutic and dyskinesia thresholds taken from Gubeila et al.^18^.

Control of PD symptoms can be achieved when blood concentrations of levodopa are maintained above the therapeutic threshold. However, when the concentration exceeds an upper threshold, motor complications, such as dyskinesia, may emerge^64^. The therapeutic and dyskinesia thresholds depend on the progression of PD, often associated with the Hoehn and Yahr (H &Y) clinical stage^18^. Figure 5a–d shows the therapeutic threshold (black dashed line) and the dyskinesia threshold (red line) in H &Y stages I–IV. As seen, the therapeutic window progressively narrows as PD advances. In stage IV patients, the therapeutic window becomes extremely narrow, making it possible to alleviate PD symptoms but not to improve motor fluctuations^65^. Therefore, for all stages except stage IV, we selected the target concentrations ($C_{target}$) as the midpoint of the therapeutic window (Table 2). For stage IV, we opted for a slightly higher concentration than the dyskinesia threshold.

The properties of the proposed MPERF were determined for different stages of PD using the procedure described in Eqs. (23–26). We present two types of MPERF: the first is a morning formulation intended to elevate levodopa blood concentrations to therapeutic levels from initially low concentrations, while the second is a late-day formulation designed to maintain concentrations within the therapeutic window and be taken 6 h after the previous dose. We assumed that the MPERF releases levodopa and carbidopa at a ratio of 4:1.

**Table 2:** The desired levodopa blood concentrations for Hoehn and Yahr clinical scale (H &Y I–H &Y IV)^18^ and the properties of the morning and late-day MPERF. The late-day pill was computed with the levodopa blood concentrations of the morning pill 6 h after administration. The properties of the MPERF were calculated with Eq. (26).

Stage	$C_{target}$	Release$_{M}$	$D_M$	Morning			Late-day		
	($\text{ng}\, \text{ml}^{-1}$)	($\text{mg}\, \text{h}^{-1}$)	(mg)	$C_{target-loading}$	$D_L$	Total	$C_{target-loading}$	$D_L$	Total
				($\text{ng}\, \text{ml}^{-1}$)	(mg)	(mg)	($\text{ng}\, \text{ml}^{-1}$)	(mg)	(mg)
I	500	14.5	76	200	24	100	500	10	86
II	550	16	84	300	38	122	550	11	95
III	750	20	107	450	58	165	700	14	121
IV	850	24	131	600	78	209	850	17	148

The properties of the morning and late-day MPERF and the parameters that were used to calculate them for the different H &Y stages of PD are shown in Table 2. To illustrate the effectiveness of the suggested MPERF, we computed levodopa blood concentrations using the model following administration of the morning MPERF, followed by a late-day MPERF after 6 h (Fig. 5). These results were then compared with the model’s predictions for the blood concentrations after administering two Sinemet CR 200 mg tablets, spaced 6 h apart.

The morning and late-day MPERF formulations consistently maintain smooth and sustained levels of levodopa, keeping them above the therapeutic threshold for a minimum of 11.5 h across all stages of PD. In patients with H &Y stages I–III (Fig. 5a–c, respectively), the MPERF effectively keeps blood concentrations below the dyskinesia threshold throughout this duration. For patients with H &Y stages IV, while the MPERF can alleviate PD symptoms, it may not provide significant relief from dyskinesia due to the narrowed therapeutic window^65^.

The predicted outcomes of the proposed MPERF demonstrate a significant improvement compared to administering two Sinemet CR tablets across all stages of PD. In patients at H &Y stages I–III, two Sinemet CR tablets maintain levodopa concentrations above the therapeutic threshold (compare solid to dashed lines in Fig. 5a–c, respectively), but the concentrations also exceed the dyskinesia threshold for at least 2 h in each tablet. This finding is consistent with Ahlskog et al.^11^ that reported at least 2.5 h of dyskinesia following administration of Sinemet CR. For patients in stage IV, the relief from PD symptoms is shorter than the period of time expected with the morning and late-day MPERF. Specifically, blood levels decrease below the lower therapeutic threshold 3 h after the first dose. Therefore, administration of Sinemet CR every 3 h is required to maintain the therapeutic effect, as previously reported^11^.

Furthermore, the proposed MPERF necessitates smaller doses of levodopa. While two Sinemet CR tablets contain 400 mg of levodopa, the morning and late-day MPERF require 186 mg, 217 mg, 286 mg, and 357 mg H &Y stages I–IV, respectively.

## Discussion

Developing a CRF that would deliver a consistent and sustained therapeutic blood concentration of levodopa proved to be a difficult task^4^. To facilitate the development of such formulation, we developed a physiologically-based mathematical model that describes the absorption of levodopa following administration in a CRF (Eqs. 1–12). Our key results: (i) levodopa is effectively absorbed throughout the entire SI (ii) the release of levodopa in the stomach causes fluctuations in the blood concentrations during the first 3 h after administration; (iii) levodopa that is released in the SI is absorbed almost immediately (iv) the release rate and dosage that extends the therapeutic duration of a formulation are contingent on the stage of PD (v) using MPERF is expected to improve the performance of levodopa CRF in all the stages of PD.

The mathematical model describes the kinetics of levodopa absorption process, and was validated by comparing its predictions to experimental results in healthy and Parkinsonian patients following administration in different CRF. The parameters that describe the physiological processes were derived from in-vivo studies^15,16,25–31^, while the release rate from the CR formulation was taken from in-vitro experiments^32,33^. Hence, the model can be used in the development process of future formulations, either by determining the advantage of new formulations, or used to analyze the data and provide additional insight to the absorption process. For example, we used the model to provide further evidence that levodopa is absorbed effectively along the entire SI.

Another potential application of the model is to facilitate individualized therapy. In principle, the model can be employed to enhance the dosing regimen of levodopa for existing commercial formulations or to personalize the $D_M$ and $D_L$ of the MPERF based on individual pharmacokinetics. Improving the dosing regimen of levodopa necessitates statistically characterizing individual parameters, such as the elimination rate from the body and those with substantial intraindividual variability, such as stomach emptying, which strongly influences blood concentration. While estimating certain parameters may not always be feasible beforehand, the model can be used to design a standard dosing regimen and assist patients in understanding how to manage irregular days (though not predicting when such days will occur). Nevertheless, estimating these parameters for every patient may be impractical. Alternatively, employing machine learning to estimate these parameters based on the patient’s clinical behavior might be feasible. This involves complementing the model presented in this study with a pharmacodynamic model of levodopa, similar to the one reported by Contin et al.^12^, and inferring the parameters from the patient’s state (e.g., using the Unified Parkinson’s Disease Rating Scale^12^). By gathering sufficient data, it may be possible to reconstruct the distribution of individual-based parameters and then employ the model to optimize the treatment.

By analyzing the model results (Fig. 4), we demonstrated that erratic gastric emptying leads to pulsatile levodopa concentrations in the blood within the first 3 h after administration. This finding is consistent with previous studies^2,30,31,34,40,41^, highlighting the significance of further elucidating the underlying mechanism.

The mathematical model also predicts that levodopa is rapidly absorbed, suggesting that releasing levodopa at a constant rate would lead to smooth and consistent concentrations in the blood, thereby improving motor fluctuations. This prediction aligns with the findings of Stocchi et al.^2^, which demonstrate that intraintestinal infusion of levodopa results in smooth and constant concentrations, leading to reduced motor complications. Another significant implication of the rapid absorption of levodopa from the SI is its limited exposure to degrading processes, such as those mediated by the microbiota, luminal enzymes, and chemical reactions^18^. As a result, the overall impact of these processes on levodopa’s bioavailability is expected to be minimal.

The erratic emptying in the presence of levodopa in the stomach, and its rapid absorption from the SI suggest that it is preferable to avoid from releasing levodopa in the stomach and release levodopa constantly along the SI. However, a single-unit enteric coated formulation, similar to the one described in a study by Flashner, et al.^32^ remains in the stomach for 1–1.5 h^25^, and possibly even longer for patients with PD, so the relief from PD symptoms will be delayed. This might pose a problem since some patients might not believe the dose worked and take another dose^5^. An alternative solution to this issue is the use of MPERF^62,66^, such as the extended-release formulation of levodopa IPX066 (Rytary)^67^. Such formulations comprised of beads (or pellets) with diameters under 2 mm with a coating that releases levodopa at different rates along the SI. Particles of such size can pass the pylorus continuously^62^, and therefore elevate the blood concentrations of levodopa shortly after the administration.

In such formulations, the residence time in the SI determines the duration a single dose can maintain therapeutic concentrations. Hence, formulations that prolong residence time in the SI, such as mucoadhesive formulations^68^, could potentially extend the therapeutic effect of a single dose. Using the model, we have determined the properties of MPERF that would achieve therapeutic concentrations approximately 30 min after administration and maintain them for approximately 6 h, with a reduced number of daily dosing and smaller doses of levodopa compared to standard levodopa or controlled-release formulations^12,69^. We note that we spaced the doses for 6 h as this reflects the residence time in the gastro-SI system. However, considering that PD may slow gastroenteric transit times, the mean residence time in PD patients may be longer, suggesting the possibility of increasing the dose to provide relief for extended periods. A better characterization of transit times in PD patients is necessary to achieve this.

One limitation of this study is that it does not consider a meal’s influence on absorption kinetics. The presence of a meal can delay stomach emptying and increase competition between the amino acids in the meal and levodopa for transport across the gut, brain, and kidneys, as described in a study by Guebila et al.^18^. The impact of meals on levodopa absorption has been studied in both experimental^31,61,64,70^, and theoretical^18^ studies.

In conclusion, we demonstrated that adjusting a CRF’s release rate can extend the therapeutic duration of a single dose of levodopa. This supports using a combination of theoretical modeling, in-vivo experiments, and in-vitro testing as a powerful tool for developing novel CR formulations.

### Supplementary Information

Supplementary Information.

## Data Availability

The data used or analyzed during the current study is available from the corresponding author Yehuda Arav on reasonable request.
