# Supplementary material for: Model-based optimization of controlled release formulation of levodopa for Parkinson’s disease
Source: Sci Rep. 2023 Sep 22;13:15869. doi: 10.1038/s41598-023-42878-5 (PMC10517026; doi:10.1038/s41598-023-42878-5)
Supplement: Supplementary file 1 — Supplementary Information. [file 41598_2023_42878_MOESM1_ESM.pdf]

Model-based optimization of controlled release formulation of levodopa for Parkinson's disease - Supplementary Information

Surface area of the SI

The inner surface of the small intestine in contact with the luminal fluid is largely increased due to three structural elements: the folds, villi, and microvilli. The calculation of the amplification as a function of the distance from the Pylorus is taken from Willman et al.[1]. Specifically, the large folds are commonly observed in the lower portion of the duodenum until approximately the middle section of the ileum. As one moves along the small intestine, these folds diminish in size and quantity. Consequently, their amplification factor decreases from 3 (between the middle duodenum and mid jejunum) to 1 (no amplification) at the distal end of the ileum, as indicated in the model. The intestinal mucosa of the gut wall is equipped with villi. From a geometric perspective, each villus can be conceptualized as a cylindrical tube with a spherical top. The average height of the villi linearly decreases from 800 μm (at the proximal end) to 500 μm (at the distal end of the small intestine). The radius of an individual villus remains nearly constant throughout the small intestine, approximately 50 μm, and their density is reportedly around 25 mm^{-2}. Regarding the amplification factor of the microvilli, a mean value of 25 was utilized (with reported interindividual variability ranging from 15 to 40).

Sinemet CR 200mg individuals

In this section, we describe the blood concentrations of healthy individuals following the administration of Sinemet CR 200mg taken from Arav et al.[2]. Figure S1 shows levodopa blood concentrations in 7 individuals following administration of Sinemet CR 200/50mg (200 mg levodopa and 50mg Carbidopa, an Aromatic L-Amino acid Decarboxylase). The individuals were non-smoking healthy male subjects (ages 18-55)[2], and fasted for at least 10 hours before administration. The concentration-time profiles of levodopa in 5 subjects (individuals 1,2,3,6,7) exhibit similar behavior. Specifically, the maximal concentrations

and double peaks were seen in the first 3 hours. The other 2 subjects (individuals 4 and 5) exhibit a delayed and slow absorption, with maximal concentrations obtained 4 to 5 hours after administration. In all subjects, the half-life time of the decay of levodopa in the blood is 1.5 hours, which is the characteristic decay rate of levodopa following concomitant administration with AADC inhibitor[3]. The occurrence of double peaks in blood concentrations following oral administration of levodopa is well documented in the literature and was attributed to an erratic gastric emptying[4–6].

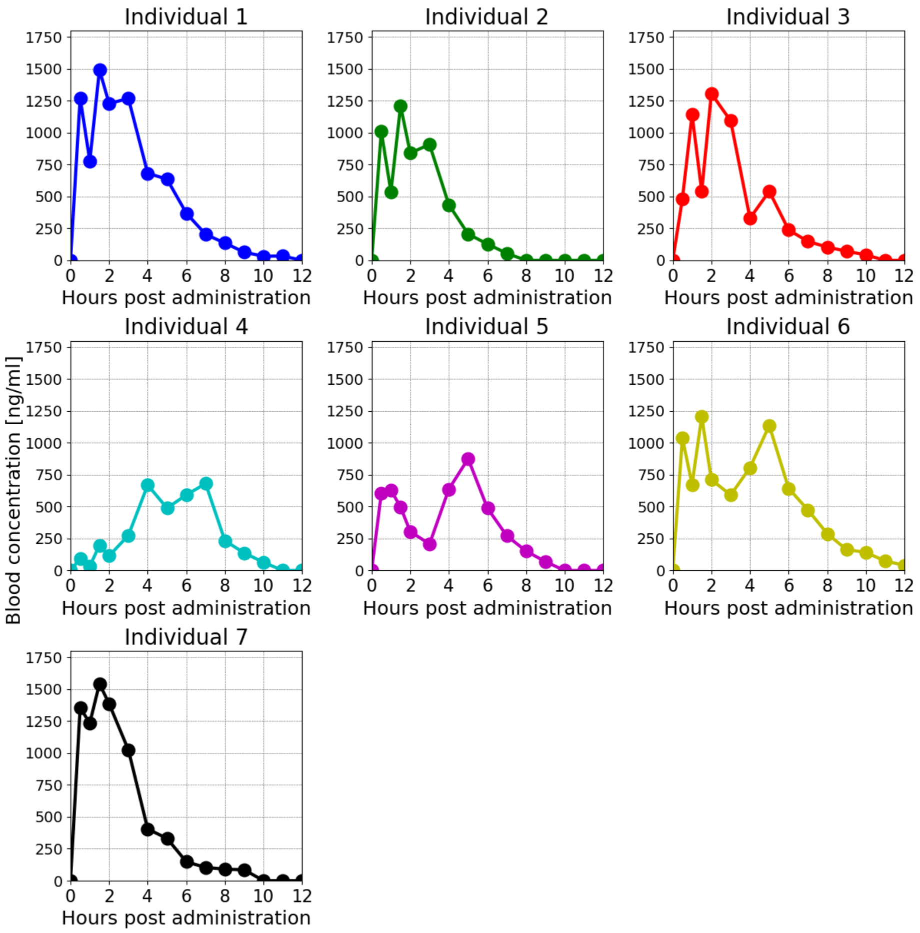

Figure S1. Experimental concentration-time profile after administering Sinemet CR 200/50mg to fasting subjects. (a-g) concentration-time profile of healthy individuals. Taken from Arav et al.[2].

These results show that the rise, peak magnitude and location, and decay of the mean levodopa blood concentrations are similar to those observed in the individuals. Therefore, we can conclude that the mean concentrations of levodopa in the blood accurately represent the kinetics of levodopa in the individuals.

Sinemet CR 100mg individuals

In this section, we describe the blood concentrations of healthy individuals following the administration of Sinemet CR 100mg taken from Arav et al.[2]. Figure S2 shows levodopa blood concentration in 8 individuals following administration of Sinemet CR 100/25mg (100mg levodopa and 25mg Carbidopa, an Aromatic L-Amino acid Decarboxylase). The individuals were non-smoking healthy male subjects (ages 18-55)[2], and fasted for at least 10 hours before administration.

In all the individuals, the peak levodopa blood concentrations are obtained within the first hour after administration. In 4 of the 8 individuals (numbers 1,4,5, and 8), the concentration-time profile exhibits a double peak, where the second peak is smaller than the first. In 7 of the 8 individuals (numbers 1,2,3,4,5,7, and 8), the concentrations decay smoothly after 2 hours. Individual 6 exhibited a small rise in the blood concentrations after 4 hours before the concentrations decayed. These results show that the rise, peak magnitude and location, and decay of the mean levodopa blood concentrations are similar to those observed in the individuals. Therefore, we can conclude that the mean concentrations of levodopa in the blood accurately represent the kinetics of levodopa in the individuals.

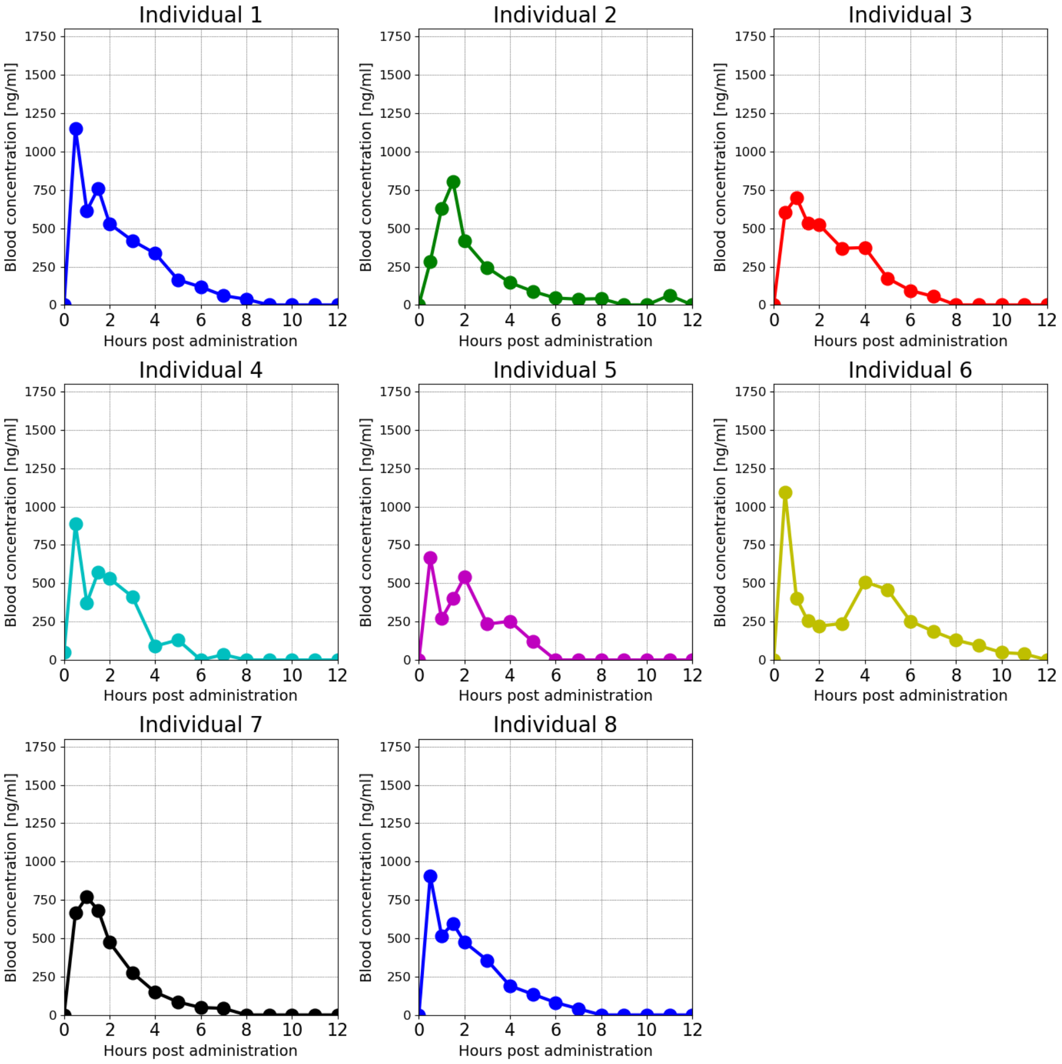

Figure S2. Experimental Concentration time profile following administration of Sinemet CR 100/50mg to healthy subjects. Taken from Arav et al.[2].

Enteric coated controlled release 200mg individuals

This section describes healthy individuals' blood concentrations after administering 200mg Enteric-coated controlled release formulation taken from Flashner et al.[7].

Figure S3 shows the concentration-time profile of the 12 individuals. Except for individual 6, the concentration-time profiles of the individuals share a close resemblance to each other. Specifically, the rise in the concentrations begins approximately 2 hours

after administration, exhibits a single peak after 4 to 6 hours, and then a decay in the concentrations.

These results show that the rise, peak magnitude and location, and decay of the mean levodopa blood concentrations are similar to those observed in the individuals. Therefore, we can conclude that the mean concentrations of levodopa in the blood accurately represent the kinetics of levodopa in the individuals.

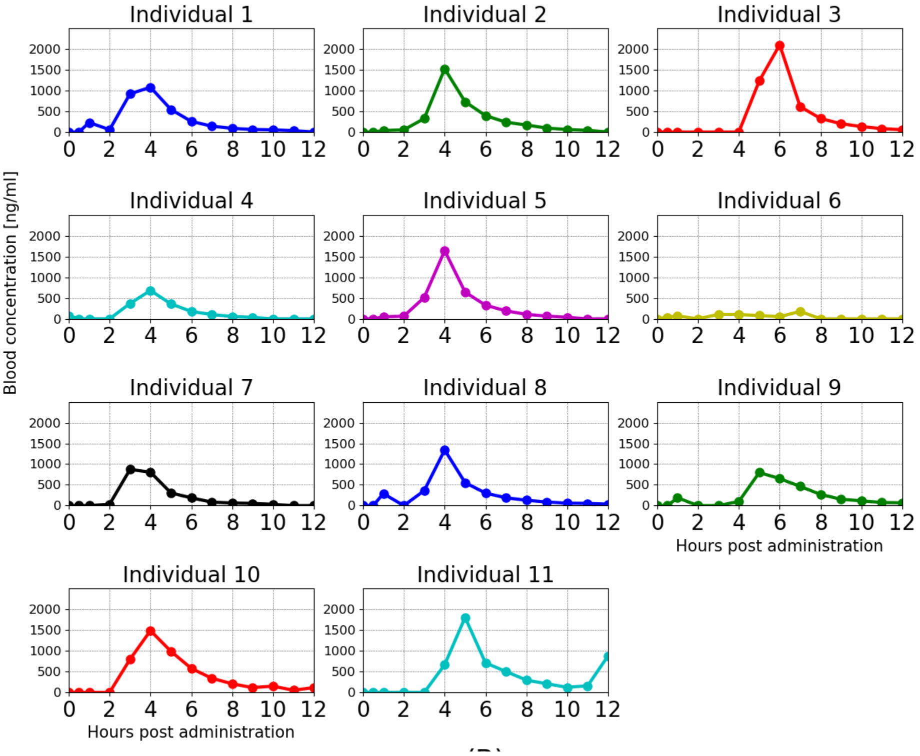

Figure S3. Experimental Concentration time profile following administration of Enteric-coated controlled release 200mg to healthy subjects. Taken from[7].

The effect of stomach emptying on levodopa blood concentrations

The mean experimental blood concentrations following administration of Sinemet CR exhibit large variability in healthy and Parkinsonian patients during the first 3 hours after administration (Figure S4). It has been suggested that large inter- and intra-

variability in the stomach emptying process. To further test this hypothesis, we conducted simulations with different parameters for stomach emptying. Specifically, we selected the stomach emptying rate, the lag duration, and the tablet's residence time in the stomach. The values examined were taken from the range reported in the literature for these parameters[6,8].

Figure S4 depicts the effect of stomach emptying parameters on levodopa blood concentrations. Large variability is observed during the first 3 hours. The stomach emptying rate and the tablet residence time determine the rise of levodopa blood concentrations and the location of the second peak, while the duration of the lag determines the depth of the double peak. Note, that the absence of lag removes the double peak (Figure S4, simulation 1).

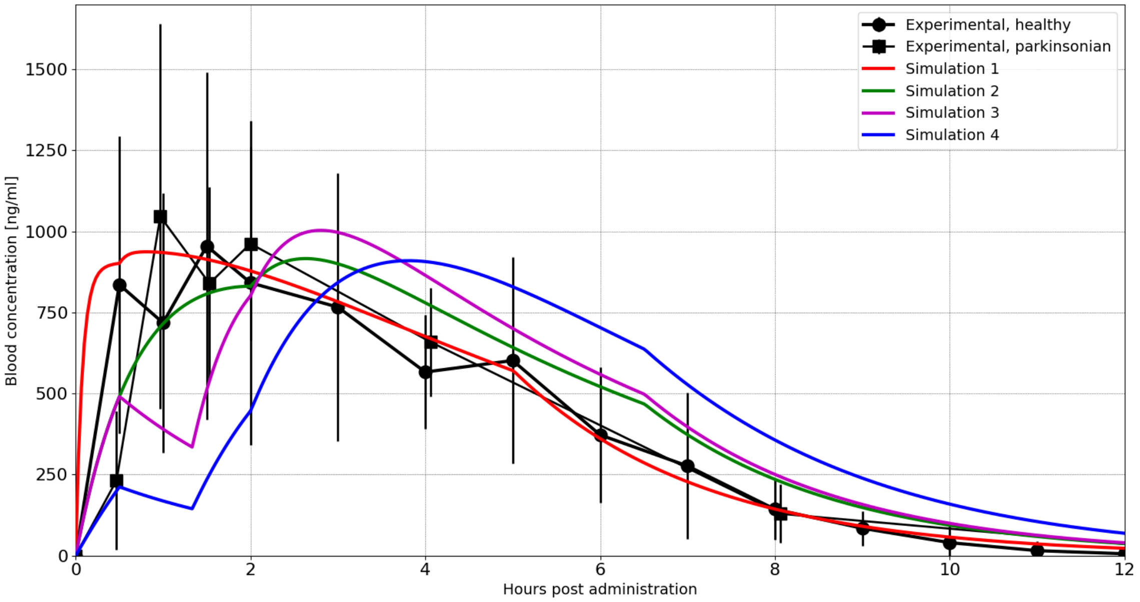

Figure S4. The effect of gastric emptying parameters on levodopa blood concentrations following Sinemet CR 200mg administration. The parameters of the simulations are given in Table S1

Table S1. The parameters used in the levodopa simulations. Parameter ranges were taken from Robinson et. al.[6] and From Davis et al.[8].

Simulation	Stomach emptying $t_{1/2}$ [min]	Emptying lag [h]	Tablet residence in Stomach [h]
1	3	0	0.5
2	26	0	2
3	26	50	2
4	75	50	2.5

References

[1] Willmann, S., Schmitt, W., Keldenich, J., Lippert, J. & Dressman, J. B. A physiological model for the estimation of the fraction dose absorbed in humans. *Journal of Medicinal Chemistry* **47**, 4022–4031 (2004).

[2] Arav, Y. *Mathematical modeling of intestinal drug absorption.* Ph.D. thesis, Hebrew university of Jerusalem (2008).

[3] Cedarbaum, J. M. Clinical Pharmacokinetics of Anti-Parkinsonian Drugs. *Clinical Pharmacokinetics* **13**, 141–178 (1987). URL http://www.ncbi.nlm.nih.gov/pubmed/3311529 http://link.springer.com/10.2165/00003088-198713030-00002.

[4] Evans, M. A., Triggs, E. J., Broe, G. A. & Saines, N. Systemic availability of orally administered L-dopa in the elderly Parkinsonian patient. *European Journal of Clinical Pharmacology* **17**, 215–221 (1980).

[5] Robertson, D. R. C. *et al.* The influence of Levodopa on gastric emptying in man. *Br j. clin pharamacol* **29**, 47–53 (1990).

[6] Robertson, D. R. *et al.* The influence of levodopa on gastric emptying in healthy elderly volunteers. *European Journal of Clinical Pharmacology* **42**, 409–412 (1992). URL https://pubmed.ncbi.nlm.nih.gov/1516606/.

[7] Flashner-Barak, M., Lerner, E. & Rosenberger, V. US Patent Application for Composition and dosage form for sustained effect of levodopa Patent Application (Application #20050113452 issued May 26, 2005) - Justia Patents Search (2005). URL `https://patents.justia.com/patent/20050113452`.

[8] Davis, S. S., Hardy, J. G. & Fara, J. W. The transit of dosage forms through the small intestine. *Gut* **27**, 886–892 (1986).
